# Neural Processing Underlying Executive Functions in Bilinguals: “Heads I Win, Tails You Lose”

**DOI:** 10.3389/fnhum.2021.710905

**Published:** 2021-08-04

**Authors:** Jesús Cespón

**Affiliations:** Basque Centre on Cognition, Brain and Language, Donostia-San Sebastián, Spain

**Keywords:** replication crisis, bilingualism, executive functions, event- related potentials, contradictory interpretations

## Introduction

Many studies have claimed bilingualism strengthens the neural mechanisms that underpin executive functions and enhances cognition in the elderly (Bialystok, 2017). Nevertheless, the field of bilingualism research has suffered from contradictory interpretations of results and many of the neural differences between monolinguals and bilinguals (in some cases, such patterns of results are difficult to interpret) have been taken as evidence for enhanced neural processing in bilinguals compared to monolinguals (de Bruin et al., inpress; Paap et al., 2015). Currently, researchers disagree regarding the existence of improved executive functioning in bilinguals compared to monolinguals—e.g., Bialystok (2017) states there is evidence for the mentioned improvements whereas Paap et al. (2015) remain skeptical.

**Figure 1:**
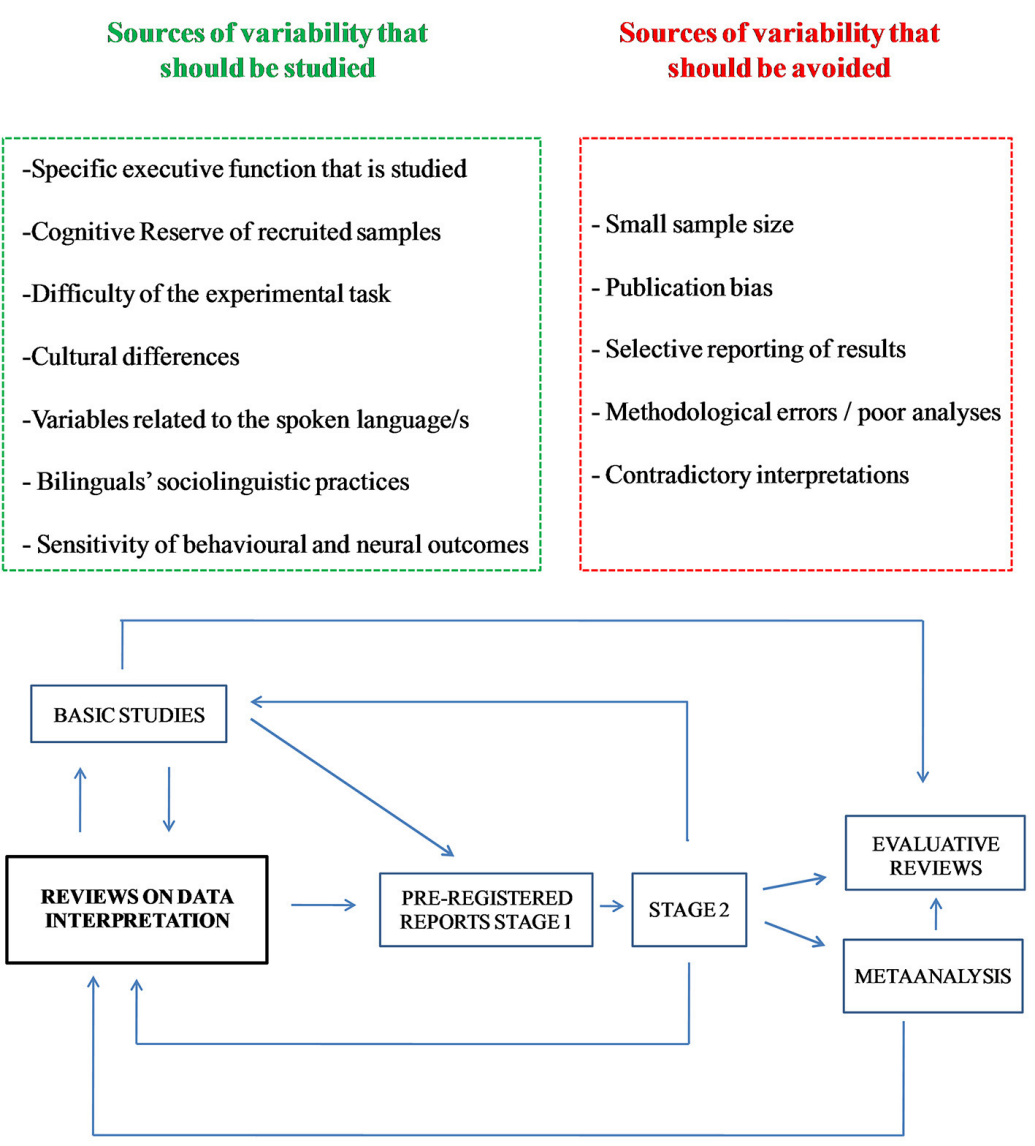
Bilingualism and executive functioning: studying results variability and improving results interpretability. Sources of results variability (top panel); how “Reviews on data interpretation” can increase the reliability and interpretability of results (bottom panel). Bidirectional interaction between different types of scientific articles will help advance research. For instance, “Reviews on data interpretation” feed the hypotheses in “Pre-registered reports, Stage 1” and, at the same time, results from “Stage 2” may modify the hypotheses formulated in “Reviews on data interpretation.” The term “basic studies” refers to research that investigates neural modulations under experimental conditions in young and/or elderly samples. Bilingualism and executive functioning: studying results variability and improving results interpretability.

In the present article, after recapping the main sources of variability in research findings (Figure 1, top panel), contradictory interpretation of results is examined. This issue highlights the importance of theoretically-grounded studies such as Cespón and Carreiras (2020), which defines what specific event-related brain potential differences between monolinguals and bilinguals should be taken to indicate enhanced bilingual neural processing during executive tasks.

## Sources of Variability in Results

A main source for the considerable variability in reported results comes from the fact that researchers have investigated different types of executive functions. Early studies stated that inhibition was the specific executive function enhanced by bilingualism. Scientists argued that this advantage resulted from bilinguals' life-long practice in inhibiting their non-target language during conversation (Bialystok et al., 2004). However, subsequent studies (e.g., Kirk et al., 2014; Antón et al., 2016) failed to replicate these early findings. Later studies claimed that bilingualism mainly enhanced attentional switching, since bilinguals need to switch attention in order to use different languages in different contexts. However, there have also been studies that failed to obtain any evidence for a bilingual advantage in switching skills (Mor et al., 2015; Ramos et al., 2017; Goldsmith and Morton, 2018). Other studies suggested that bilingualism enhances working memory (Grundy and Timmer, 2017) or monitoring (Costa et al., 2009). Nevertheless, negative results were also frequently reported (e.g., Kirk et al., 2014; Lukasik et al., 2018).

There are several variables that could lead to ceiling effects in executive control tasks and obscure real differences between monolinguals and bilinguals. For instance, tasks with low levels of difficulty could mask a bilingual advantage in executive functions due to ceiling effects in performance (Bialystok et al., 2014; Kuipers and Westphal, 2021). This could also happen if executive control tasks are administered to young adults, who are at the top of their performance (Bialystok et al., 2008; Ware et al., 2020). Also, recruiting monolinguals and bilinguals with high cognitive reserve (CR) may blur differences in executive functions since other CR factors (e.g., a high level of education) could strength cognition in monolinguals (Bialystok et al., 2016) by improving functional compensatory mechanisms (Cespón et al., 2018).

Variables related to spoken language/s such as age of acquisition, the number of languages spoken, the degree of similarity between these languages, and the relative degrees of proficiency a speaker has in each of their languages may also modulate any potential bilingual enhancement in executive functioning (Bialystok, 2017). Sociolinguistic practices could also influence which executive processes are enhanced by bilingualism (Hofweber et al., 2020). Differences between cultures may constitute another source of differences in executive functioning (Samuel et al., 2018; Treffers-Daller et al., 2020). Overall, the samples recruited for studies have differed widely in terms of the variables listed above (Cespón and Carreiras, 2020), possibly explaining a substantial portion of the variability in results. Importantly, this variability may be reduced by analyzing neurophysiological measures, which are more sensitive than behavioral correlates to differences between monolinguals and bilinguals (Grundy et al., 2017a).

## Methodological Problems

Button et al. (2013) estimated that only 8% to 31% of studies in neuroscience are properly powered. The field of research investigating the relationship between bilingualism and the neural mechanisms underlying executive functions is no exception. As noted by Cespón and Carreiras (2020), sample sizes for event-related potential (ERP) studies typically include only 20–25 participants per group, the standard sample size in ERP literature. This low statistical power may partly explain the often inconsistent findings (Baker, 2016).

Publication bias has affected bilingualism research (de Bruin et al., 2015) like other fields of psychology (Kühberger et al., 2014). Specifically, the proportion of negative to positive results in studies on the relationship between bilingualism and executive functioning was higher in conference abstracts than in scientific journals (de Bruin et al., 2015); this suggests scientific journals are biased to accept studies that show positive results.

Selective reporting is another factor that contributes to the low replicability of reported results; researchers may run analyses until they find something that matches their predictions, as pointed out by Chambers (2017). Selective reporting may be difficult to identify but can sometimes be detected. For instance, it is unclear why Fernández et al. (2014) in a replication of their previous study (Fernández et al., 2013) used different methods to analyse the N200 ERP component. A more worrying issue is the presence of objective methodological errors that could have been avoided through a careful peer-review process, as detailed by Paap et al. (2020).

## Contradictory Interpretation of Results

Previous research has already suggested that pre-registered reports and multi-center studies are appropriate measures to avoid publication bias and selective reporting of results (e.g., Paap et al., 2020). Preventing biased interpretations of results and agreeing on how specific patterns of data should be interpreted is another important issue. In this context, theoretical reviews—such as Cespón and Carreiras (2020), which provides guidelines on how to interpret specific patterns of data in line with basic research findings—will contribute to establishing well-founded hypotheses for future studies and to interpreting upcoming research results in an unbiased way. Figure 1 (bottom panel) shows how suitable relationships among different types of studies could help advance research in a reliable manner.

Cespón and Carreiras (2020) reviewed the main ERP modulations (specifically, N200, P300, N450, and error related negativity) used to investigate enhanced executive functioning in bilingual research. They also outline how these ERP modulations have been interpreted in basic psychophysiological studies outside the field of bilingualism. Using this approach, the authors established which ERP differences between monolinguals and bilinguals can be reliably interpreted as demonstrating enhanced neural processing. They argued that faster ERP latencies in group “x” compared to group “y” indicate more efficient processing by group “x” even in the absence of behavioral differences. However, ERP amplitudes are usually more difficult to interpret than ERP latencies (Cespón and Carreiras, 2020); reliable interpretation would require a well-established theoretical framework or evidence for significant correlations between ERP amplitudes and behavioral performance.

A number of studies have investigated ERP differences in the neural processes involved in executive functioning in monolinguals and bilinguals (Kousaie and Phillips, 2012, 2017; Fernández et al., 2013, 2014; Coderre and van Heuven, 2014; Moreno et al., 2014; Heidlmayr et al., 2015; Morales et al., 2015; Barac et al., 2016; Grundy et al., 2017b; López-Zunini et al., 2019; Morrison et al., 2019). All of these ERP studies—except Kousaie and Phillips (2012)—have claimed a bilingual advantage in neural correlates underlying executive tasks; nevertheless, only three studies (Barac et al., 2016; Kousaie and Phillips, 2017; Morrison et al., 2019) demonstrated that their results matched predictions based on basic research, as discussed in Cespón and Carreiras (2020).

There is widespread misinterpretation of N200 amplitude modulations. The fact that N200 amplitudes are higher in bilinguals than monolinguals performing executive tasks has erroneously been taken as evidence for enhanced neural processing in bilinguals (Fernández et al., 2013, 2014; Moreno et al., 2014; Morales et al., 2015). Research in other fields has demonstrated that increased fronto-central N200 amplitudes relate to greater effort and increased neural deployment of inhibitory processes (Jodo and Kayama, 1992; Kopp et al., 1996; Falkenstein et al., 1999; Heil et al., 2000; Liotti et al., 2000; Bokura et al., 2001; Clayson and Larson, 2011). In this broader research context, it seems highly implausible that increased N200 amplitudes constitute a neural signature of enhanced neural processing during bilingual performance of executive control tasks. The results obtained by Moreno et al. (2014) illustrate this point. These authors found that bilinguals had larger N200 amplitudes than monolinguals during the performance of a Go/No-Go task. Moreover, this N200 amplitude was also larger in monolingual non-musicians than monolingual musicians. Thus, according to the interpretation of N200 amplitudes in the bilingual literature, we would have to deduce that being a musician impairs neural processing related to executive functioning. However, this interpretation is highly implausible since being a musician is considered a factor that contributes to CR (Román-Caballero et al., 2018; Andrews et al., 2021).

There are some ERP studies that have offered partial or inconclusive evidence for enhanced executive neural processing in bilinguals relative to monolinguals. Kousaie and Phillips (2017) observed a bilingual advantage in some tasks but not in other tasks that measured similar processes. Another study reported a behavioral bilingual advantage in attentional switching, but the underlying neural mechanisms could not be clearly identified (López-Zunini et al., 2019). Most ERP studies have focused on the classical N200 and P300 components (Cespón and Carreiras, 2020). Importantly, future research should also investigate other ERP correlates of executive processes that are thought to be enhanced by bilingualism, such as the negativity central contralateral and negativity posterior contralateral, which relate to inhibition and attentional shifting, respectively (Cespón et al., 2020).

The existence of contradictory interpretations in studies investigating relationships between bilingualism and executive functions was also indicated outside ERP literature (Paap et al., 2015; García-Pentón et al., 2016). Research based on magnetic resonance imaging (MRI) has sometimes interpreted opposite patterns of results as evidence for a bilingual advantage. For example, some studies on the healthy elderly have claimed that increased white matter integrity of the corpus callosum in bilinguals demonstrated that they showed better structural preservation than monolinguals (Luk et al., 2011; Pliatsikas et al., 2015). In contrast, other studies have claimed that reduced white matter in the corpus callosum of bilinguals relative to monolinguals demonstrated enhanced CR in bilinguals; the argument is that bilinguals are able to match the performance of monolinguals despite impairments in corpus callosum structure (Gold et al., 2013). Studies focused on the healthy elderly using functional MRI have concluded that higher connectivity in prefrontal areas indicates that bilinguals have greater brain capacity than monolinguals (Grady et al., 2015), whereas Berroir et al. (2017) claimed that lower connectivity in prefrontal areas in elderly bilinguals relative to monolinguals reveals more efficient processing in the bilingual brain. A number of the interpretations of neural differences between monolinguals and bilinguals resemble the logic of “Heads I win, tails you lose,” with opposite patterns of results interpreted as beneficial neural modulations related to bilingualism.

The development of theoretically-grounded reviews in basic science (e.g., Cespón and Carreiras, 2020), which may be labeled “Reviews on data interpretation” (see Figure 1, bottom panel), could reduce the type of contradictory interpretations mentioned in the previous paragraph by establishing how specific data patterns will be interpreted beforehand. If there is disagreement on how to interpret specific results, this will highlight relevant issues that require further basic research.

Also, these “Reviews on data interpretation” could provide solid hypotheses for pre-registered studies. If these pre-registered studies become a common practice within the field, over the long-term, meta-analyses could be conducted on pre-registered studies. This would represent an interesting approach given the higher methodological quality of pre-registered compared to conventional studies and the possibility of correcting for publication bias simply by taking withdrawn pre-registrations into account.

In conclusion, by clarifying how specific patterns of data should be interpreted, taking the key sources of variability into consideration, and avoiding the methodological errors reviewed here, progress can be made. These are essential steps to clarify whether and how specific experimental conditions lead to enhanced neural processing underlying executive functioning in bilinguals.

## Author Contributions

The author confirms being the sole contributor of this work and has approved it for publication.

## Conflict of Interest

The author declares that the research was conducted in the absence of any commercial or financial relationships that could be construed as a potential conflict of interest.

## Publisher's Note

All claims expressed in this article are solely those of the authors and do not necessarily represent those of their affiliated organizations, or those of the publisher, the editors and the reviewers. Any product that may be evaluated in this article, or claim that may be made by its manufacturer, is not guaranteed or endorsed by the publisher.

## References

[B1] Andrews E. Eierud C. Banks D. Harshbarger T. Michael A. Rammell C. (2021). Effects of lifelong musicianship on white matter integrity and cognitive brain reserve. Brain Sci. 11:67. 10.3390/brainsci11010067 33419228 PMC7825624

[B2] Antón E. García Y. F. Carreiras M. Duñabeitia J. A. (2016). Does bilingualism shape inhibitory control in the elderly? J. Men. Lang. 90, 147–160. 10.1016/j.jml.2016.04.007

[B3] Baker M. (2016). 1,500 scientists lift the lid on reproducibility. Nature 533, 452–454. 10.1038/533452a 27225100

[B4] Barac R. Moreno S. Bialystok E. (2016). Behavioral and electrophysiological differences in executive control between monolingual and bilingual children. Child Dev. 87, 1277–1290. 10.1111/cdev.12538 27133956 PMC4939124

[B5] Berroir P. Ghazi-Saidi L. Dash T. Adrover-Roig D. Benali H. Ansaldo A. I. (2017). Interference control at the response level: functional networks reveal higher efficiency in the bilingual brain. J. Neurolinguistics 43(Pt A), 4–16. 10.1016/j.jneuroling.2016.09.007

[B6] Bialystok E. (2017). The bilingual adaptation: how minds accommodate experience. Psychol. Bull. 143, 233–262. 10.1037/bul0000099 28230411 PMC5324728

[B7] Bialystok E. Abutalebi J. Bak T. H. Burke D. M. Kroll J. F. (2016). Aging in two languages: implications for public health. Aging Res. Rev. 27, 56–60. 10.1016/j.arr.2016.03.003 26993154 PMC4837064

[B8] Bialystok E. Craik F. I. Klein R. Viswanathan M. (2004). Bilingualism, aging, and cognitive control: evidence from the Simon task. Psychol. Aging 19, 290–303. 10.1037/0882-7974.19.2.290 15222822

[B9] Bialystok E. Luk G. Craik F. (2008). Cognitive control and lexical access in younger and older bilinguals. J. Exp. Psychol. Learn. Mem. Cogn. 34, 859–873. 10.1037/0278-7393.34.4.859 18605874

[B10] Bialystok E. Poarch G. Luo L. Craik F. (2014). Effects of bilingualism and aging on executive function and working memory. Psychol. Aging 29, 696–705. 10.1037/a0037254 25244487 PMC4274603

[B11] Bokura H. Yamaguchi S. Kobayashi S. (2001). Electrophysiological correlates for response inhibition in a Go/NoGo task. Clin. Neurophysiol. 112, 2224–2232. 10.1016/S1388-2457(01)00691-5 11738192

[B12] Button K. S. Ioannidis J. P. A. Mokrysz C. Nosek B. A. Flint J. Robinson E. S. J.. (2013). Power failure: why small sample size undermines the reliability of neuroscience. Nat. Rev. Neurosci. 14, 365–376. 10.1038/nrn3475 23571845

[B13] Cespón J. Carreiras M. (2020). Is there electrophysiological evidence for a bilingual advantage in neural processes underlying executive functions? Neurosci. Biobehav. Rev. 118, 315–330. 10.1016/j.neubiorev.2020.07.030 32758515

[B14] Cespón J. Hommel B. Korsch M. Galashan D. (2020). The neurocognitive underpinnings of the Simon effect: an integrative review of current research. Cogn. Affect Behav. Neurosci. 20, 1133–1172. 10.3758/s13415-020-00836-y 33025513

[B15] Cespón J. Miniussi C. Pellicciari M. C. (2018). Interventional programmes to improve cognition during healthy and pathological ageing: cortical modulations and evidence for brain plasticity. Ageing Res. Rev. 43, 81–98. 10.1016/j.arr.2018.03.001 29522820

[B16] Chambers C. (2017). The Seven Deadly Sins of Psychology: A Manifesto for Reforming the Culture of Scientific Practice. Princeton, NJ: Princeton University Press. 10.1515/9781400884940

[B17] Clayson P. E. Larson M. J. (2011). Conflict adaptation and sequential trial effects: support for the conflict monitoring theory. Neuropsychologia 49, 1953–1961. 10.1016/j.neuropsychologia.2011.03.023 21435347

[B18] Coderre E. L. van Heuven W. J. B. (2014). Electrophysiological explorations of the bilingual advantage: evidence from a Stroop task. PLoS ONE 9:e103424. 10.1371/journal.pone.0103424 25068723 PMC4113364

[B19] Costa A. Hernández M. Costa-Faidella J. Sebastián-Gallés N. (2009). On the bilingual advantage in conflict processing: now you see it, now you don't. Cognition 113, 135–149. 10.1016/j.cognition.2009.08.001 19729156

[B20] de Bruin A. Barbara T. Della Sala S. (2015). Cognitive advantage in bilingualism: an example of publication bias? Psychol. Sci. 26, 99–107. 10.1177/0956797614557866 25475825

[B21] de Bruin A. Dick A. S. Carreiras M. (inpress). Clear theories are needed to interpret differences: perspectives on the bilingual advantage debate. Neurobiol. Lang. 1–46. 10.1162/nol_a_00038 PMC10158573 37214628

[B22] Falkenstein M. Hoormann J. Hohnsbein J. (1999). ERP components in the go/no-go tasks and their relation to inhibition. Acta Psychol. 101, 267–291. 10.1016/S0001-6918(99)00008-6 10344188

[B23] Fernández M. Acosta J. Douglass K. Doshi N. Tartar J. L. (2014). Speaking two languages enhances an auditory but not a visual neural marker of cognitive inhibition. AIMS Neurosci. 1, 145–157. 10.3934/Neuroscience.2014.2.145

[B24] Fernández M. Tartar J. L. Padron D. Acosta J. (2013). Neurophysiological marker of inhibition distinguishes language groups on a non-linguistic executive function test. Brain Cogn. 83, 330–336. 10.1016/j.bandc.2013.09.010 24141240

[B25] García-Pentón L. Fernández García Y. Costello B. Duñabeitia J. A. Carreiras M. (2016). The neuroanatomy of bilingualism: how to turn a hazy view into the full picture. Lang. Cogn. Neurosci. 31, 303–327. 10.1080/23273798.2015.1068944

[B26] Gold B. T. Johnson N. F. Powell D. K. (2013). Lifelong bilingualism contributes to cognitive reserve against white matter integrity declines in aging. Neuropsychologia 51, 2841–2846. 10.1016/j.neuropsychologia.2013.09.037 24103400 PMC3856701

[B27] Goldsmith S. F. Morton J. B. (2018). Sequential congruency effects in monolingual and bilingual adults: a failure to replicate Grundy et al. (2017). Front. Psychol. 9:2476. 10.3389/fpsyg.2018.02476 30618921 PMC6297870

[B28] Grady C. L. Luk G. Craik F. I. Bialystok E. (2015). Brain network activity in monolingual and bilingual older adults. Neuropsychologia 66, 170–181. 10.1016/j.neuropsychologia.2014.10.042 25445783 PMC4898959

[B29] Grundy J. G. Anderson J. A. E. Bialystok E. (2017a). Neural correlates of cognitive processing in monolinguals and bilinguals. Ann. N. Y. Acad. Sci. 1396, 183–201. 10.1111/nyas.13333 28415142 PMC5446278

[B30] Grundy J. G. Chung-Fat-Yim A. Friesen D. C. Mak L. Bialystok E. (2017b). Sequential congruency effects reveal differences in disengagement of attention for monolingual and bilingual young adults. Cognition 163, 42–55. 10.1016/j.cognition.2017.02.010 28273520 PMC5398762

[B31] Grundy J. G. Timmer K. (2017). Bilingualism and working memory capacity: a comprehensive meta-analysis. Second Lang. Res. 33, 325–340. 10.1177/0267658316678286

[B32] Heidlmayr K. Hemforth B. Moutier S. Isel F. (2015). Neurodynamics of executive control processes in bilinguals: evidence from ERP and source reconstruction analyses. Front. Psychol. 6:821. 10.3389/fpsyg.2015.00821 26124740 PMC4467069

[B33] Heil M. Osman A. Wiegelmann J. Rolke B. Hennighausen E. (2000). N200 in the Eriksen-task: inhibitory executive process? J. Psychophysiol. 14, 218–225. 10.1027//0269-8803.14.4.218

[B34] Hofweber J. Marinis T. Treffers-Daller J. (2020). How different code-switching types modulate bilinguals' executive functions: a dual control mode perspective. Biling. Lang. Cogn. 23, 909–925. 10.1017/S1366728919000804

[B35] Jodo E. Kayama Y. (1992). Relation of negative ERP component to response inhibition in a go/no-go task. Electroencephalogr. Clin. Neurophysiol. 82, 477–482. 10.1016/0013-4694(92)90054-L 1375556

[B36] Kirk N. W. Fiala L. Scott-Brown K. C. Kempe V. (2014). No evidence for reduced Simon cost in elderly bilinguals and bidialectals. J. Cogn. Psychol. 26, 640–648. 10.1080/20445911.2014.929580 25264481 PMC4164011

[B37] Kopp B. Rist F. Mattler U. (1996). N200 in the flanker task as a neurobehavioral tool for investigating executive control. Psychophysiology 33, 282–294. 10.1111/j.1469-8986.1996.tb00425.x 8936397

[B38] Kousaie S. Phillips N. A. (2012). Conflict monitoring and resolution: are two languages better than one? evidence from reaction time and event-related brain potentials. Brain Res. 1446, 71–90. 10.1016/j.brainres.2012.01.052 22356886

[B39] Kousaie S. Phillips N. A. (2017). A behavioural and electrophysiological investigation of the effect of bilingualism on aging and cognitive control. Neuropsychologia 94, 23–35. 10.1016/j.neuropsychologia.2016.11.013 27876508

[B40] Kühberger A. Fritz A. Scherndl T. (2014). Publication bias in psychology: a diagnosis based on the correlation between effect size and sample size. PLoS ONE 9:e105825. 10.1371/journal.pone.0105825 25192357 PMC4156299

[B41] Kuipers J. R. Westphal K. H. (2021). Auditory processing and high task demands facilitate the bilingual executive control advantage in young adults. J. Neurolinguistics 57:100954. 10.1016/j.jneuroling.2020.100954

[B42] Liotti M. Woldorff M. G. Perez R. Mayberg H. S. (2000). An ERP study of the temporal course of the Stroop color-word interference effect. Neuropsychologia 38, 701–711. 10.1016/S0028-3932(99)00106-2 10689046

[B43] López-Zunini R. A. Morrison C. Kousaie S. Taler V. (2019). Task switching and bilingualism in young and older adults: a behavioral and electrophysiological investigation. Neuropsychologia 133:107186. 10.1016/j.neuropsychologia.2019.107186 31513809

[B44] Luk G. Bialystok E. Craik F. I. Grady C. L. (2011). Lifelong bilingualism maintains white matter integrity in older adults. J. Neurosci. 31, 16808–16813. 10.1523/JNEUROSCI.4563-11.2011 22090506 PMC3259110

[B45] Lukasik K. M. Lehtonen M. Soveri A. Waris O. Jylkkä J. Laine M. (2018). Bilingualism and working memory performance: evidence from a large-scale online study. PLoS ONE 13:e0205916. 10.1371/journal.pone.0205916 30388118 PMC6214526

[B46] Mor B. Yitzhaki-Amsalem S. Prior A. (2015). The joint effect of bilingualism and ADHD on executive functions. J. Atten. Disord. 19:527e541. 10.1177/1087054714527790 24681900

[B47] Morales J. Yudes C. Gómez-Ariza C. J. Bajo M. T. (2015). Bilingualism modulates dual mechanisms of cognitive control: evidence from ERPs. Neuropsychologia 66, 57–69. 10.1016/j.neuropsychologia.2014.11.014 25448864

[B48] Moreno S. Wodniecka Z. Tays W. Alain C. Bialystok E. (2014). Inhibitory control in bilinguals and musicians: event related potential (ERP) evidence for experience-specific effects. PLoS ONE 9:e941690094169. 10.1371/journal.pone.0094169 24743321 PMC3990547

[B49] Morrison C. Kamal F. Taler V. (2019). The influence of bilingualism on working memory event-related potentials. Biling. Lang. Cogn. 22, 191–199. 10.1017/S1366728918000391 30646813

[B50] Paap K. R. Johnson H. A. Sawi O. (2015). Bilingual advantages in executive functioning either do not exist or are restricted to very specific and undetermined circumstances. Cortex 69, 265–278. 10.1016/j.cortex.2015.04.014 26048659

[B51] Paap K. R. Mason L. Zimiga B. Ayala-Silva Y. Frost M. (2020). The alchemy of confirmation bias transmutes expectations into bilingual advantages: a tale of two new meta-analyses. Q. J. Exp. Psychol. (Hove) 73, 1290–1299. 10.1177/1747021819900098 31931663

[B52] Pliatsikas C. Moschopoulou E. Saddy J. D. (2015). The effects of bilingualism on the white matter structure of the brain. Proc. Natl. Acad. Sci. 112, 1334–1337. 10.1073/pnas.1414183112 25583505 PMC4321232

[B53] Ramos S. Fernández García Y. Antón E. Casaponsa A. Duñabeitia J. A. (2017). Does learning a language in the elderly enhance switching ability? J. Neurolinguistic. 43, 39–48. 10.1016/j.jneuroling.2016.09.001

[B54] Román-Caballero R. Arnedo M. Triviño M. Lupiáñez J. (2018). Musical practice as an enhancer of cognitive function in healthy aging. A systematic review and meta-analysis. PLoS ONE 13:e0207957. 10.1371/journal.pone.0207957 30481227 PMC6258526

[B55] Samuel S. Roehr-Brack K. Pak H. Kim H. (2018). Cultural effects rather than a bilingual advantage in cognition: a review and an empirical study. Cogn. Sci. 42, 2313–2341. 10.1111/cogs.12672 30136441

[B56] Treffers-Daller J. Ongun Z. Hofweber J. Korenar M. (2020). Explaining individual differences in executive functions performance in multilinguals: the impact of code-switching and alternating between multicultural identity styles. Front Psychol. 11:561088. 10.3389/fpsyg.2020.561088 33192829 PMC7644971

[B57] Ware A. T. Kirkovski M. Lum J. A. G. (2020). Meta-analysis reveals a bilingual advantage that is dependent on task and age. Front. Psychol. 11:1458. 10.3389/fpsyg.2020.01458 32793026 PMC7394008

